# A Randomized Comparison of Bougie-Assisted and TracheoQuick Plus Cricothyrotomies on a Live Porcine Model

**DOI:** 10.1155/2017/4215159

**Published:** 2017-12-19

**Authors:** Tomas Henlin, Pavel Michalek, Tomas Tyll, Ondrej Ryska

**Affiliations:** ^1^Department of Anesthesia and Intensive Medicine, First Medical Faculty, Charles University and University Military Hospital, U Vojenske Nemocnice 1200, 169 02 Prague, Czech Republic; ^2^Department of Anesthesia and Intensive Medicine, First Medical Faculty, Charles University and General University Hospital, U Nemocnice 2, 128 08 Prague, Czech Republic; ^3^Department of Anaesthesia, Antrim Area Hospital, Bush Road, Antrim BT41 2RL, UK; ^4^Institute of Animal Physiology and Genetics, Academy of Sciences of the Czech Republic (AS CR), Rumburska 89, 277 21 Libechov, Czech Republic

## Abstract

**Objectives:**

Cricothyrotomy is a rescue procedure in “cannot intubate, cannot oxygenate” scenarios where other methods of nonsurgical airway management have failed. We compared 2 cuffed cricothyrotomy sets, bougie-assisted cricothyrotomy (BACT) and novel percutaneous TracheoQuick Plus, on a live porcine model in a simulated periarrest situation.

**Methods:**

Thirty-four anesthetized minipigs were randomly allocated into two groups: BACT technique (*n* = 17) and TracheoQuick Plus (*n* = 17). The primary outcome was duration of cricothyrotomy while secondary outcomes were total success rate, number of attempts, location of incision, changes in heart rate, oxygen saturation, and the incidence of complications.

**Results:**

BACT was significantly faster than TracheoQuick Plus cricothyrotomy, with a median time of 69 sec (IQR 56–85) versus 178 sec (IQR 152–272). The total success rate was without difference. 94% of BACT was performed successfully on the first attempt, while in the TracheoQuick Plus group, it was only 18% (*P* < 0.001). Trauma to the posterior tracheal wall was observed once in the BACT group and 5 times in the TracheoQuick Plus group. Oxygen saturation was significantly higher in the BACT group both during and after the procedure.

**Conclusions:**

BACT is superior to TracheoQuick Plus cricothyrotomy on a live animal model.

## 1. Introduction

Emergency infraglottic approach to the airway via the cricothyroid membrane is indicated in any situation where other more conventional methods either fail or are not feasible due to disturbed orofacial anatomy [1, 2]. The incidence of emergency cricothyrotomy varies widely in the literature, from 0.002% in the operating room [3] to 10.9% in the prehospital setting [4]. In some major trauma cases, an emergency cricothyrotomy may be an alternative method of emergency airway management to tracheal intubation [5]. The Tactical Combat Casualty Care (TCCC) algorithm for field trauma emergencies recommends surgical cricothyrotomy as a “second” step approach in the management of the obstructed airway [6]. Various commercial or home-made sets are available for this procedure. Recent meta-analyses or systematic reviews do not favor one particular technique of performing cricothyrotomy over others due to insufficient data [1, 7]. Bougie-assisted cricothyrotomy (BACT) was described as a 3-step procedure in 2007 as a simple, quick, and effective procedure of emergency airway management [8]. Most randomized studies comparing different cricothyrotomy techniques were performed on human or animal cadaveric models or on simulators. In the present study, we proposed to use a live porcine model with pharmacologically induced respiratory depression presenting as irregular breathing (gasping) in order to simulate realistic conditions of emergency airway management, including monitoring of vital functions. The aim of our study was to compare BACT to a novel, cuffed, commercially available set for cricothyrotomy, TracheoQuick Plus® (Teleflex Medical Ltd., Ireland).

## 2. Material and Methods

The study was performed with written approval from the Ethical Committee of the Academy of Sciences of the Czech Republic (10/2013). The study was performed in accordance with the Czech legislation related to animal studies (law 10/1992) and respecting the ARRIVE protocol.

### 2.1. Study Design

The study was designed to be prospective and randomized. The animals were randomized 2 h prior to enrollment using sealed envelopes. Two operators with experience in emergency medicine and surgical airway management performed all of the procedures. Both operators had practiced each procedure 20 times on manikins and 5 times on cadavers prior to commencement of this study.

### 2.2. Laboratory Animals

In this study, live minipigs weighing 35–50 kg were used for all experiments. All the animals were properly farmed. Minipigs from other experiments (experimental model of sepsis) already intended for postmortem examination were used. Animal handling was performed in accordance with the European Directive for the Protection of Vertebrate Animals Used for Experimental and Other Scientific Purposes (86/609/EU). All experiments were closely supervised by a qualified veterinary physician.

### 2.3. Equipment and Procedures

**Figure 1:**
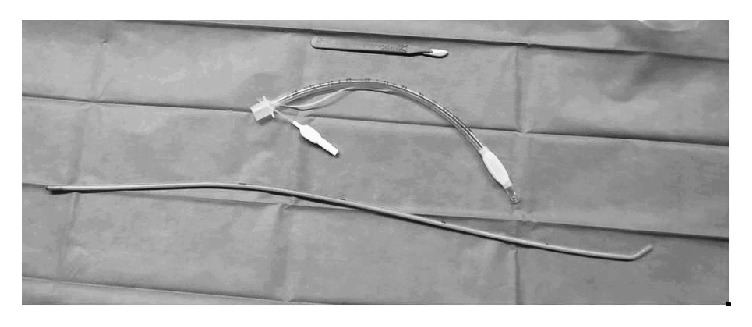
Equipment for BACT procedure: scalpel, cuffed 6.5 mm ID endotracheal tube, and gum-elastic bougie.

Two cricothyrotomy sets were used for the purpose of this study. For the BACT technique, a surgical scalpel with a number 20 blade, a 60 cm long gum-elastic bougie (Venn Reusable Tracheal Tube Introducer, Smith Medical International Ltd., UK), and a 6.5 mm ID cuffed endotracheal tube (Smith Medical, Ashton, UK) were used (Figure 1). The procedure was performed in 3 steps: a “T” shape was stabbed through the skin, subcutaneous tissue, and cricothyroid membrane (step 1); the gum-elastic bougie was inserted through the membrane until resistance was felt (step 2); the tracheal tube was railroaded over the bougie into the trachea (step 3). Following insertion, the cuff of the endotracheal tube was inflated and the tube was connected to the breathing circuit.

**Figure 2:**
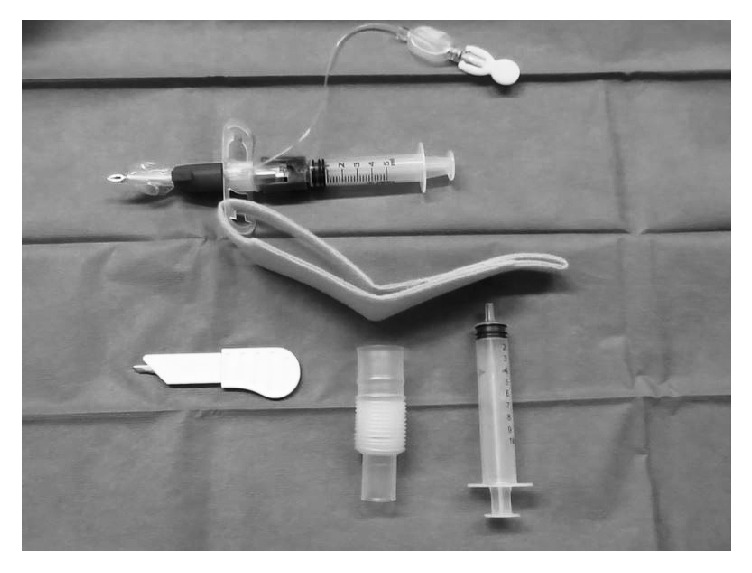
TracheoQuick Plus cricothyrotomy kit.

The TracheoQuick Plus Coniotomy Tube Rüsch set (Teleflex Medical Europe Ltd., Ireland) (Figure 2) contains a short surgical scalpel, cuffed cricothyrotomy tube with an internal diameter of 4.0 mm, flexible tube extension, and one-way syringe. When performing cricothyrotomy with TracheoQuick Plus, the larynx was immobilized and a vertical skin incision was made with the scalpel. The cricothyroid membrane was subsequently punctured with the set. Once the tracheal lumen was reached with tip of the needle, the safety device was removed. At this point, the device was angled caudally and the cricothyrotomy tube was slid into the tracheal lumen. The cuff was then inflated and the device connected to the breathing circuit. In total, 4 attempts were allowed in each group. In the case of failure, confirmed by an absence of an etCO_2_ waveform on capnography, the pigs were subsequently intubated. The procedures were performed in the operating veterinary theater without the use of artificial light.

### 2.4. Anesthesia and Measurements

General anesthesia was induced with an intramuscular mixture of anesthetics. A mixture of tiletamine and zolazepam at a dose of 2 mg/kg and ketamine 2 mg/kg with xylazine 0.4 mg/kg was used. Following this, monitoring of oxygen saturation and ECG was initiated, and an intravenous cannula was inserted. Propofol was given intravenously until the onset of irregular breathing. At this point, an operator commenced skin incision. Following successful cricothyrotomy and device placement, the tube was connected to the anesthetic circuit, etCO_2_ was monitored, and the lungs were ventilated with 100% oxygen for 3 minutes.

### 2.5. Outcomes

The primary outcome measure for this study was time to successful device placement. This time was measured from the operator starting the procedure until the first effective controlled breath as seen on capnography.

Secondary outcomes involved the total success rate of the procedure (visible effective breath on capnography), number of attempts, first attempt success rate, changes in heart rate during and after the procedure as measured on the ECG monitor, and changes in oxygen saturation measured by a pulse oximeter placed on the tongue of the pig. Oxygen saturation was measured at incision time, immediately before cricothyrotomy device placement, and after 1 min of artificial ventilation with 100% O_2_ through the device. Intraoperative complications such as massive venous or arterial bleeding, subcutaneous emphysema, and difficulties with anatomical orientation were recorded.

The location of the incision and traumatic complications were evaluated on postmortem examination. All animals were euthanized 2 hours after their cricothyrotomies.

### 2.6. Sample Size and Statistical Analysis

We used the results from our previously published pilot study of BACT [9] to determine the sample size for this trial. Based on these results, a mean procedure time of 72 sec (SD ± 30) was used and a meaningful difference between the groups was chosen as 30 sec. The sample size was calculated as minimum of 16 animals in each arm with 80% power and 5% alpha using a 2-tailed test. A total number of 34 animals were finally randomized in order to compensate for potential drop-outs.

Before definitive analysis, all data were tested for normal distribution using the Shapiro–Wilk test. Based on their distribution, the results were analyzed using either parametric (Fisher's exact text, Student's *t*-test) or nonparametric (Mann–Whitney *U* test) statistical tests. The statistical software program InStat (GraphPad Software Inc., La Jolla, USA) was used for analysis.

## 3. Results

**Figure 3:**
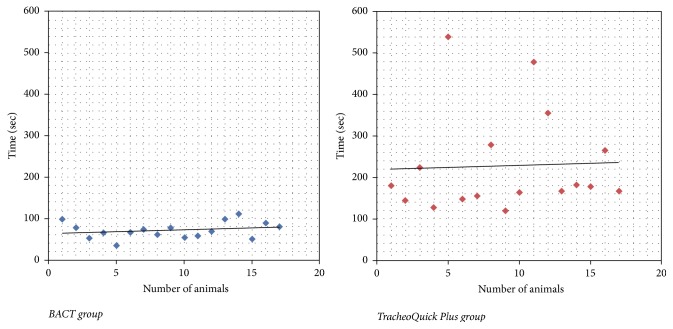
Histogram of distribution of the procedure duration between the groups.

**Figure 4:**
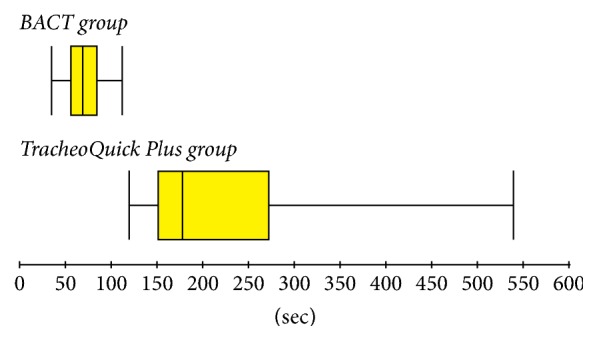
Box plot of procedure duration (primary outcome).

**Figure 5:**
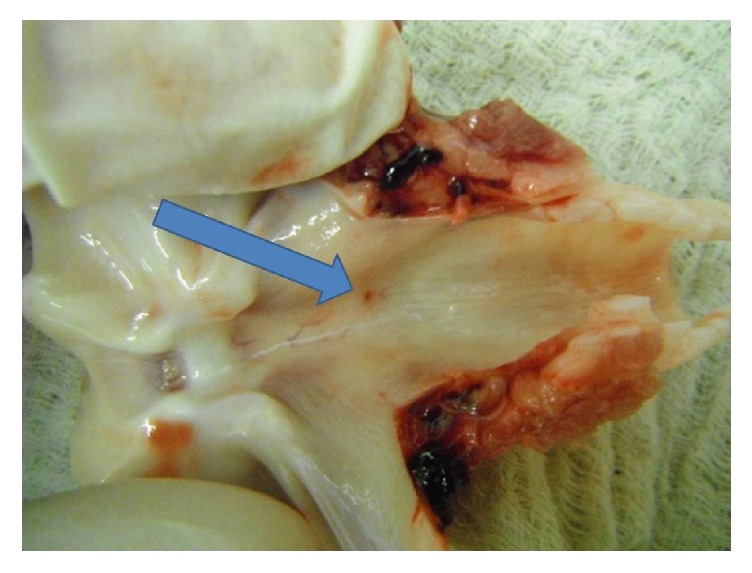
Trauma to the posterior tracheal wall (arrow) on postmortem examination.

In total, 34 cricothyrotomies were performed (17 in the BACT group, 17 in the TracheoQuick Plus group). Baseline parameters, such as weight, did not differ significantly between the groups. The procedure time was significantly shorter in the BACT group (median 68 sec, IQR 56.5–85 sec) than in the TracheoQuick Plus group (median 178 sec, IQR 151.5–272.5 sec); *P* < 0.001 (Figures 3 and 4). All cricothyrotomies in the BACT group were successful, with the majority being performed on the first attempt (Table 1). In the TracheoQuick Plus group, 14 procedures (82.3%) were successful, with only 3 (17.6%) being performed on the first attempt (Table 1). All incisions in the BACT group were located within the cricothyroid membrane, while in the TracheoQuick Plus group, 1 puncture was located between the first and second tracheal ring and another 3 were into the paratracheal tissue. There were no significant differences in the baseline profile, postprocedural values, or intraoperative changes in heart rates between the groups (Table 2). Intraprocedural and postprocedural oxygen saturation were significantly higher in the BACT group (Table 2). There was 1 case of trauma to the posterior tracheal wall in the BACT group recorded during postmortem examination at the level of the first tracheal ring and at 5 o'clock on the pars membranacea of the trachea (Figure 5). In total, 5 cases of trauma to the posterior tracheal wall (29.4%) were observed in the TracheoQuick Plus group. We did not observe any significant venous or arterial bleeding in any animal, and we did not see any case of pneumothorax, pneumomediastinum, or presence of subcutaneous emphysema. There was 1 case of hypoxic cardiac arrest in the TracheoQuick Plus group (5.9%); all other animals survived the procedure.

## 4. Discussion

The results of this prospective randomized trial showed that BACT is significantly faster method of emergency cricothyrotomy than the commercially available percutaneous cricothyrotomy set TracheoQuick Plus. Apart from shorter time, BACT was also significantly more successful at the first attempt.

Percutaneous or surgical cricothyrotomy is a life-saving procedure in “cannot intubate, cannot oxygenate” (CICO) situations. There have been continuing debates in the literature about the most appropriate and most successful technique of emergency front of neck access. A recent systematic review analyzed all human, animal, and simulation studies related to emergency cricothyrotomy [1]. The authors concluded that no technique of percutaneous, Seldinger, or surgical cricothyrotomy showed significant superiority over another technique, but the quality of studies varied and sample sizes were mostly low. Data obtained from NAP4 highlighted the fact that surgical techniques are potentially more successful and effective than needle cricothyrotomies [3].

BACT was first described by MacIntyre et al. in 2007 as a quick surgical cricothyrotomy performed by military physicians under difficult conditions such as lack of light [8]. Few studies have compared BACT with other cricothyrotomy techniques. Hill et al. compared BACT with a standard surgical cricothyrotomy on an anesthetized sheep model [10]. BACT showed a similarly low failure rate and was also significantly faster than the standard technique.

The 3-step bougie-assisted method was considered a faster, more successful, and safer method than conventional surgical cricothyrotomy using a high-fidelity airway manikin [11]. On the other hand, the Portex-cuffed percutaneous cricothyrotomy device, similar to the TracheoQuick Plus kit, was associated with a faster insertion time than BACT using a dissected porcine larynx model [12]. However, the success rate was higher and the complication rate lower in the BACT group prior to intensive training. The TracheoQuick Plus device has not been tested yet and the only data available are derived from similar cuffed devices such as Quicktrach II or the Portex cricothyrotomy kit. Noncuffed percutaneous devices showed significantly shorter insertion times than the cuffed kit Quicktrach II on a manikin [13]. Four different cricothyrotomy techniques on human unembalmed cadavers found higher success rates and fewer complications using a surgical approach when compared to puncture/percutaneous techniques [14]. Three different cuffed percutaneous kits were compared to surgical cricothyrotomy in another simulation study [15]. The Quicktrach II and Melker sets performed better than a surgical approach, and the Portex kit was associated with the highest failure rate.

Most studies comparing devices for emergency cricothyrotomies have been performed on simulators, dissected animal models, or cadavers.

The main problem during cadaveric and manikin studies of surgical cricothyrotomies is a lack of reality in regard to the biomechanical properties of the tissues, as well as an absence of potential complications, such as swelling or local bleeding from the subcutaneous or thyroid vessels [16]. Setting up a prospective randomized study comparing 2 different methods of emergency surgical airway management in humans is extremely difficult due to the low incidence of the procedure, ethical reasons, and diversity of clinical scenarios.

### 4.1. Study Limitations

This study has several limitations. First, the anatomy of the porcine larynx is slightly different from that of humans. While the cartilaginous structures are grossly similar, the porcine skin is significantly thicker, the distance between skin and trachea is longer in pigs, and palpation of the porcine cricothyroid membrane is more difficult than it is in humans [17, 18].

Second, the procedures were performed in almost ideal conditions. The pigs were anesthetized, with only a small reaction to the incision, lying in the supine position on the floor with the theaters illuminated by natural light. In real life, emergency cricothyrotomies are often performed under significant stress, with poor or no light and on patients who may be moving or struggling.

Third, all cricothyrotomies were performed by physicians experienced in airway management including performing a surgical airway procedure. The results may have been different using inexperienced operators, paramedics, or medical students [19].

In addition, landmark techniques have been found to be inaccurate in locating the cricothyroid membrane [19, 20], and ultrasound guidance, even in emergency situations, might facilitate device placement [21].

## 5. Conclusions

Based on this animal experiment performed using a live porcine model, the bougie-assisted cricothyrotomy was shown to be a faster and less traumatic method for emergency surgical airway management than cricothyrotomy using the TracheoQuick Plus device. BACT appears to be superior to the TracheoQuick Plus device in a live porcine model. Future studies should be directed toward the use of ultrasound in emergency surgical airways [22], performance of BACT under endoscopic control [23], and the setting up of a large prospective multicenter trial comparing BACT and percutaneous methods of emergency cricothyrotomy on emergency patients.

## Figures and Tables

**Table 1:** Procedure time, success rate, number of attempts, and complications. Data are presented as median, range (), and interquartile range [].

	BACT	TracheoQuick Plus	*P*
*n*	17	17	
Procedure time (sec)	69	178	
	(35–112) [56.5–85]	(120–539) [151.5–272.5]	<0.001^*∗*^
Success rate (*n*, %)	17 (100)	14 (82.3)	0.22
Number of attempts (*n*)	1 (1-2) [1-1]	2 (1–4) [2-3]	<0.001^*∗*^
First time success (*n*, %)	16 (94.1)	3 (17.6)	<0.001^*∗*^
Complications total (*n*, %)	1 (5.9)	8 (47.1)	0.0167^*∗*^
(i) Posterior wall trauma	1 (5.9)	5 (29.4)	0.175
(ii) Paratracheal puncture	-	3 (17.6)	0.227
(iii) Rupture of cartilage	-	-	
(iv) Bleeding	-	-	
(v) Pneumothorax	-	-	
(vi) Pneumomediastinum	-	-	

^*∗*^Statistically significant.

**Table 2:** Changes in heart rate and oxygen saturation during and after the cricothyrotomy. Data are presented as mean (SD).

	BACT	TracheoQuick Plus	*P*
*n*	17	17	
Heart rate (bpm)			
(i) Prior to procedure	58 (7.4)	57 (6.4)	0.676
(ii) After device insertion	88 (8.5)	92 (24)	0.52
Oxygen saturation (spO_2_) (%)			
(i) Prior to procedure	69 (10.9)	68 (10.6)	0.788
(ii) After device insertion	66 (9.9)	56 (16.5)	0.039^*∗*^
(iii) 1 min after insertion	94 (2.1)	81 (25.7)	0.046^*∗*^

^*∗*^Statistically significant.

## Abbreviations

BACT: Bougie-assisted cricothyrotomy
CICO: Cannot intubate, cannot oxygenate
ECG: Electrocardiography
NAP: National Audit Project
TCCC: Tactical Combat Casualty Care.
